# Prolonged Gestation

**Published:** 1881-03

**Authors:** 


PROLONGED GESTATION. C. A. Wilcox, M D.—Mrs C., whose menstrual flow commenced March 12, 1880, and continued as usual six days, gave birth January 11 to her fifth child, two hundred and ninety-six days after the disappearance of the menses, is positive she felt motions some days previous to July 26th. The labor was natural and recovery good. The child, a girl, weighed nine and one-half pounds, the largest of the five, and at two weeks looked as old as other children of a month. Mrs. C., while perfectly regular, has previously observed a full period of five weeks.—*Boston Medical and Surgical Journal.*

---

DR. HAMMOND's daughter, the Marquise de Lanza, has just completed a novel, to be published by the Putnam's, the plot of which turns on the idea of double consciousness. The heroine, while in the "second state," engages herself to be married and when she recovers her normal condition has forgotten all about it. Dr. Hammond will write a preface to the book upon the subject of double consciousness.—*Boston Medical and Surgical Journal.*

